# LungNet22: A Fine-Tuned Model for Multiclass Classification and Prediction of Lung Disease Using X-ray Images

**DOI:** 10.3390/jpm12050680

**Published:** 2022-04-24

**Authors:** F. M. Javed Mehedi Shamrat, Sami Azam, Asif Karim, Rakibul Islam, Zarrin Tasnim, Pronab Ghosh, Friso De Boer

**Affiliations:** 1 Department of Software Engineering, Daffodil International University, Dhaka 1207, Bangladesh; javedmehedicom@gmail.com (F.M.J.M.S.); zarrint25@gmail.com (Z.T.); 2 College of Engineering, IT and Environment, Charles Darwin University, Casuarina, NT 0909, Australia; asif.karim@cdu.edu.au (A.K.); friso.deboer@cdu.edu.au (F.D.B.); 3 Department of Computer Science and Engineering, Daffodil International University, Dhaka 1207, Bangladesh; rakibulislam9200.cse@gmail.com; 4 Department of Computer Science (CS), Lakehead University, 955 Oliver Rd, Thunder Bay, ON P7B 5E1, Canada; pghosh1@lakeheadu.ca

**Keywords:** LungNet22, X-ray image, VGG16, optimizer, CNN, lung disease, deep learning

## Abstract

In recent years, lung disease has increased manyfold, causing millions of casualties annually. To combat the crisis, an efficient, reliable, and affordable lung disease diagnosis technique has become indispensable. In this study, a multiclass classification of lung disease from frontal chest X-ray imaging using a fine-tuned CNN model is proposed. The classification is conducted on 10 disease classes of the lungs, namely COVID-19, Effusion, Tuberculosis, Pneumonia, Lung Opacity, Mass, Nodule, Pneumothorax, and Pulmonary Fibrosis, along with the Normal class. The dataset is a collective dataset gathered from multiple sources. After pre-processing and balancing the dataset with eight augmentation techniques, a total of 80,000 X-ray images were fed to the model for classification purposes. Initially, eight pre-trained CNN models, AlexNet, GoogLeNet, InceptionV3, MobileNetV2, VGG16, ResNet 50, DenseNet121, and EfficientNetB7, were employed on the dataset. Among these, the VGG16 achieved the highest accuracy at 92.95%. To further improve the classification accuracy, LungNet22 was constructed upon the primary structure of the VGG16 model. An ablation study was used in the work to determine the different hyper-parameters. Using the Adam Optimizer, the proposed model achieved a commendable accuracy of 98.89%. To verify the performance of the model, several performance matrices, including the ROC curve and the AUC values, were computed as well.

## 1. Introduction

Lung disease is categorized as the third leading cause of mortality globally [1], with an estimated total of five million deaths per year [2]. As a vital respiratory organ, the lung is not only prone to infection from components but externally as well. Lung disease manifests due to various forms of air pollution, microbiological attack, chemical intake, or physical sickness. One of the most alarming lung diseases in recent times is COVID-19. COVID-19 is detected by identifying upper respiratory tract and lung infection accompanied by pneumonia and a cold-like syndrome. The disease was first explored in Wuhan, China [3]. COVID-19 causes the air sacs in the lungs to fill with fluids leaking from the blood vessels, resulting in difficulty in breathing. Among some very common lung diseases are pneumonia and pneumothorax. Pneumothorax occurs when air escapes between the lung and the chest wall of the patient, causing the lung to completely collapse. On the other hand, pneumonia is an acute respiratory infection that causes the alveoli in the lung to fill with pus or fluid [4]. Abnormal growth in the lung indicates a lung tumor. If the growth is larger than 3 cm, it is called a mass. However, if the growth is less than 3 cm in diameter, it is known as a nodule. A benign nodule is not cancerous; however, a malignant nodule not only spreads to other parts of the body but also causes lung cancer. Effusion refers to a build-up of fluid. An excess amount of fluid builds up outside the lung and within the pleural layers [5]. Pulmonary fibrosis occurs due to damage and scars in the lung; thick and stiff tissues grow, making it laborious to breathe. Tuberculosis damages the lung by creating a large cavity in the lung and enlarging bronchiectasis. Lung opacity is identified as an area in the lung damaged by intraparenchymal hemorrhage. 

Medical X-ray imaging is a universal diagnosis technology for disease diagnosis. Using X-ray, the internal physical structure of organs and bones can be detected [6]. X-rays are valuable diagnosis techniques that have been utilized for years by experts to diagnose fractures, some cancers, pneumonia, dental issues, and other conditions. In severe circumstances, Computed Tomography (CT) may provide a succession of body scans that are then combined to create a three-dimensional X-ray picture analyzed by a computer. However, a normal X-ray is quicker, simpler, less expensive, and less invasive than a CT scan [7]. In the majority of instances, X-rays are insufficient to provide a diagnosis; CT scans are often required to confirm the diagnosis [8]. Multiple CT scans may be required for diseases that develop rapidly, but this is exceedingly costly, time-demanding, and may potentially be harmful to the patient as both of them use radiation [9].

As a result, there is a critical demand for an artificial intelligence system capable of effectively detecting chest-related illnesses [10,11]. The research includes applying deep learning to create predictions about medical pictures by extracting information such as spatial rotation and shape from the photos. CNNs have been important in the extraction of features and in the learning of patterns that allowed prediction [12]. AI has matured to the point that it can be incorporated with cutting-edge machine learning and deep learning algorithms in a variety of disciplines, including health [7], biometrics [13], agriculture [14], cloud computing [15], and renewable energy [16]. Numerous advantages are associated with AI technology in medicine. For example, rural locations and third-world countries often lack specialized physicians capable of providing the necessary treatment [17]; artificial intelligence technologies support them in obtaining the necessary medical treatment.

Additionally, this kind of research will benefit the healthcare industry significantly as professionals will be able to utilize it to support their diagnoses; additionally, it may be operated by those with no medical training. Even patients might benefit from this technology [18,19]. All of this is done to mitigate the specialist’s load, given that there are only a few of them, by focusing them only on the X-rays marked as problematic by the prediction tools. Moreover, they significantly greatly reduce a doctor’s subjective decisions, accelerate diagnosis, and reliably identify details that the human vision may miss [20]. 

In our study, X-ray images of the chest were collected from multiple sources. The datasets were collected from databases such as GitHub, Kaggle, and NIH Clinical Center [21,22,23,24,25,26,27,28,29,30,31,32,33,34,35,36]. The collection is composed of multiple lung-disease X-ray images that include COVID-19, Effusion, Lung Opacity, Mass, Nodule, Pulmonary Fibrosis, Pneumonia, Pneumothorax, and Tuberculosis, in addition to the Controlled class. The disease’s features may be easily and quickly detected using deep learning models, allowing for a more rapid and accurate diagnosis. 

The contributions of the paper are as follows:

1. The raw chest X-ray data for the study is collected from multiple data sources, merged into a collective dataset. To ensure all the data are in the same scale, preprocessing is performed.
2. Autoencoder is employed to denoise the noisy images. For removing annotation in the data, the EnsNet method is used.
3. CLAHE enhancement methods are used to increase the quality of the X-ray images, and the Green Fire Blue filtering technique is applied to improve the image properties and make them more identical to the model.
4. Augmentation is performed to balance each class of the dataset by upsampling the classes with fewer data.
5. Eight pre-trained models, namely AlexNet, GoogLeNet, InceptionV3, MobileNetV2, VGG16, ResNet 50, DenseNet121, and EfficientNetB7 are evaluated to determine the highest-performing model.
6. A fine-tuned LungNet22 is proposed based on VGG16 architecture.
7. To justify the model’s accuracy, the precision, recall, specificity, and f1-score are calculated from the confusion matrix. The loss function is also used to evaluate the performance of the models.
8. For the visual description of the model’s outcome, Grad-CAM is produced.
9. The proposed model is evaluated by computing the ROC curve and the AUC value.

**Figure 1:**
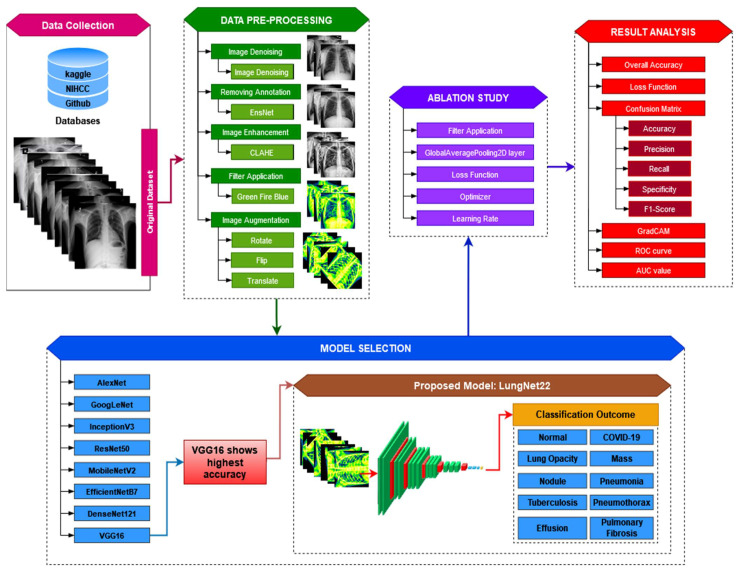
Operation of the proposed LungNet22 model for multiclass classification of lung disease.

The operating process of the proposed study is presented in Figure 1.

The rest of the paper is organized in the following manner. Section 2 summarizes the prior research on lung disease diagnosis in multiple datasets, using deep learning with the models’ accuracy scores. Section 3 discusses the collection, pre-processing, and preparation of the datasets prior to feeding the model. Section 4 covers the transfer learning models that were chosen, and their parameters, and the proposed model (LungNet22) with the ablation study by applying different parameters. The result is discussed in Section 5. Section 6 summarizes the study’s findings and compares those to other related research. Section 7 highlights the research findings and makes recommendations for further research.

## 2. Literature Review

The capacity of machine learning algorithms, particularly that of deep learning, to identify anomalies in X-ray images has gained popularity in recent years. Artificial intelligence is being used in medical research to facilitate diagnosis, and some of the studies have shown positive and accurate findings. The strategies employed by past researchers to handle lung-related disorders by utilizing artificial intelligence and deep neural networks are explored here. 

A multiclass classification of the deep learning model is suggested in [37] for identifying COVID-19, pneumonia, and lung cancer using chest X-ray and CT images. The efficiency of four architectures, which are a combination of VGG19, ResNet152V2 with CNN, GRU, and Bi-GRU, is examined the accuracy of the VGG19 + CNN model was 98.05%. The authors in [38] present a novel, hybrid deep learning framework called VGG Data STN, with CNN, VDSNet that combines with VGG, data augmentation, and a spatial transformer network (STN) with CNN. The other models implemented in the study are Vanilla Gray, Vanilla RGB, Hybrid CNN + VGG, and a modified Capsule Network. The proposed VDSNet model has a validation accuracy of 73%. This study [39] provided an accurate technique of classifying COVID-19, pneumonia, and the normal class, utilizing the CNN classification method with the histogram-oriented gradient (HOG) feature-extraction methods. A public X-ray dataset has been used to train and test the proposed CNN model. The model is validated by the use of a 10-fold cross-validation and metric confusion. 

To classify and forecast chest X-rays for various lung diseases, the authors in [40] recommended utilizing the proposed model UCMobN. The model is constructed by modifying MobileNetV2 architecture. Atelectasis, Consolidation, Edema, Effusion, Emphysema, Fibrosis, Infiltration, and Mass are among the 10 lung disorders included in the dataset. In the study [41], the authors provide a CNN-based model for automatically detecting pleural effusion in chest X-rays. The research relies on X-ray scans of patients with pleural effusion and those in a healthy state. An accuracy of 95% in the classifying of the data was found using the CNN model in this examination. The authors in [42] verify a deep CNN technique termed Decompose, Transfer, and Compose (DeTraC), which is capable of handling any anomalies in the image dataset. The approach employs a variety of CNN models, including AlexNet, VGG19, ResNet, GoogleNet, and SqueezeNet to classify data with an accuracy of 97.53%. 

Another article [43] proposes a method for augmenting synthetic data in three deep CNN models in order to diagnose 14 chest-related disorders. DenseNet121, InceptionResNetV2, and ResNet152V2 are the models used. To identify abnormalities in X-ray scans, the suggested models were trained for multiclass classification. The authors in [44] employed the Mask R-CNN approach on the X-ray dataset in order to categorize patients with and without the COVID-19 infection. Five-fold cross-validation was used to train the Mask R-CNN for 100 epochs. The authors suggest an architecture for the COVID-aid model based on the DarkCovidNet architecture for COVID-19 and pneumonia detection in [45]. This model has 19 convolutional layers and six max-pooling layers. The authors present a CNN-based classifier for pneumonia diagnosis and validate the numbers to choose the optimum model for the job depending on the specific characteristics in [46]. To X-ray images, the research applies the VGG16, VGG19, NasNetMobile, ResNet152V2, and InceptionResNetV2 models. 

Another paper [47] makes use of many state-of-the-art CNN models, DenseNet201, ResNet50V2, and InceptionV3 for detecting COVID-19 patients using chest X-ray scans. Initially, the models are separately taught to produce self-contained forecasts and then integrated using weighted average ensembling to predict a class value. In [48], using chest X-ray images as inputs, an automated CNN model is constructed and suggested to classify COVID-19 into four severity classes: COVID-19 (Mild), COVID-19 (Moderate), COVID-19 (Severe), and COVID-19 (Critical). The model produced an average accuracy of 95.52%. Almost all of the hyper-parameters in the CNN are tuned automatically by the grid search optimizer. 

The authors in [49] proposed two algorithms for COVID-19 detection, including DNN techniques based on the fractal characteristic of data and CNN methods based on lung images. The classification results indicate that the provided CNN architecture is more accurate (93.2%) than the DNN technique (83.4%). Deep learning techniques, the fine-tuning of pre-trained CNNs, and the end-to-end training of a built CNN model were utilized in this research [50] to detect COVID-19 and normal chest X-rays. Pre-trained deep CNN models were employed for deep feature extraction. An SVM classifier was utilized in conjunction with several kernel functions to classify the deep features. Additionally, the fine-tuning technique employed the previously pre-trained deep CNN models. 

The authors of this article [51] develop a Multi-scale Adaptive Residual Neural Network (MARnet) for the purpose of classifying chest X-ray scans of lung illnesses. To improve the model, the authors extract image characteristics and cross-transfer the information retrieved from the residual block and the information extracted from the adaptive structure to a separate layer, avoiding the residual structure’s reducing influence on the adaptive function. The accuracy gained by the literature investigations is summarized in Table 1.

## 3. Datasets

**Figure 2:**
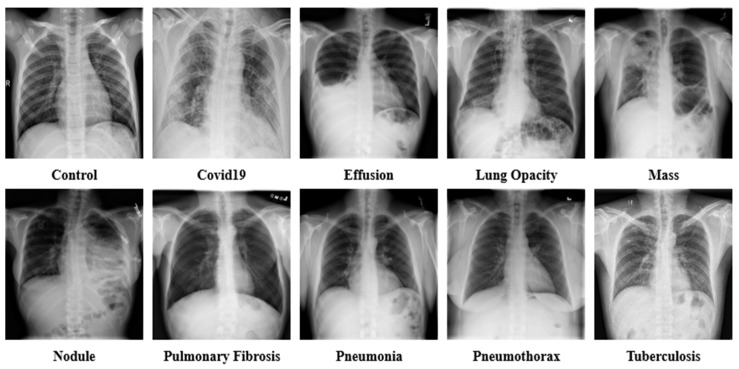
Sample dataset of the ten classes, including Control, COVID-19, Effusion, Lung Opacity, Mass, Nodule, Pulmonary Fibrosis, Pneumonia, Pneumothorax, and Tuberculosis.

In the study, X-ray images of chests were collected from multiple sources. The datasets were collected from databases such as GitHub, Kaggle, and NIH Clinical Center [21,22,23,24,25,26,27,28,29,30,31,32,33,34,35,36]. The collective dataset is a combination of 16 datasets and contains 85,105 frontal chest X-ray images. The collection consists of 10 classes of X-ray images that include COVID-19 (15,660 images), Effusion (13,501 images), Lung Opacity (7179 images), Mass (5603 images), Nodule (6201 images), Pulmonary Fibrosis (3357 images), Pneumonia (9878 images), Pneumothorax (6870 images), and Tuberculosis (3184 images), in addition to the Controlled (13,672 images) class. Table 2 contains the information of the sources of data collected for each class. The age frequency of the dataset is between 1 and 89 years. The data of both male and female patients are included in the dataset. The study is conducted to identify lung disease irrespective of age and gender. A sample of the raw dataset is presented in Figure 2. 

### 3.1. Dataset Preprocessing

The X-ray data are gathered from a variety of sources, due to the fact that the CNN model used for classification needs clean, enhanced, and balanced picture data. To feed the model with a high-quality picture, image-preparation and image-balancing techniques are utilized. This section discusses in depth the image-processing methods.

#### 3.1.1. Image Denoising

Autoencoder is a reasonable solution because of its implementation in denoising, which has a lot of potential for the extraction of features and data-component identification, which are the early stages before entering further into imaging analysis and processing. The Denoising Autoencoder (DAE) acquires the input parameters during picture reconstruction, leading to an overall improvement in latent representational extraction [52]. 

As part of the training of the model, denoising is a smart option as it preserves input info (input encode) and tries to eliminate noise from the auto-encoder input. The DAEs have been demonstrated to be edge detectors for natural picture patches and bigger stroke detectors for digit images, respectively [53]. Finally, DAEs outperform standard denoising filters because they can be adjusted to the input, while traditional filters are not data-specific. The autoencoder consists of an encoder and a decoder at a high level. “Autoencoder” is a term that refers to the automated functions of these two sections. The encoder reduces the dimensionality of the input signal to a more compressed latent state, while the decoder reverses the encoder’s work on the decoded output to recreate the original picture. The latent state of an image cannot be reconstructed by traditional autoencoders. Another variant of this is to delete portions of the input rather than adding noise to the input, in order for the model to learn to forecast the original picture. 

The dataset (image size: 224 × 224 pixel) has been used to implement the DAE. The CNN is used in this approach. Due to its efficacy in capturing spatial details, the CNN is the chosen neural network for image dataset analysis. The DAEs procedure begins with the dataset being loaded and the pixel values being normalized. The random noise is then applied to the train and test sets through the function “np.random.noise”. Thus, the noisy picture will be utilized as the feed for the encoder and the principal images as the output for training/testing the model. The formula “F(X)=Y” is essentially used, where *X* is the source noise-free picture, and *Y* is indeed the noisy image. 

During training, a loss function similar to the root mean squared error (RMSE) is set, and the network estimates and tries to decrease the losses among the denoised images (reconstructed pictures) from the decoder and the raw picture in each iteration. From Equation (1), the validation loss is measured by considering the network output ($y^{i}$) to the actual output (yi), and as the network advances on inputs, this loss may be predicated on a loss function, where ℒ is the individual loss calculated by taking into account the difference between the predicted and the actual output.

(1) $$J=1/N\sum N_{i}=1ℒ\;\left(y^{i},y_{i}\right)\;$$

**Figure 3:**
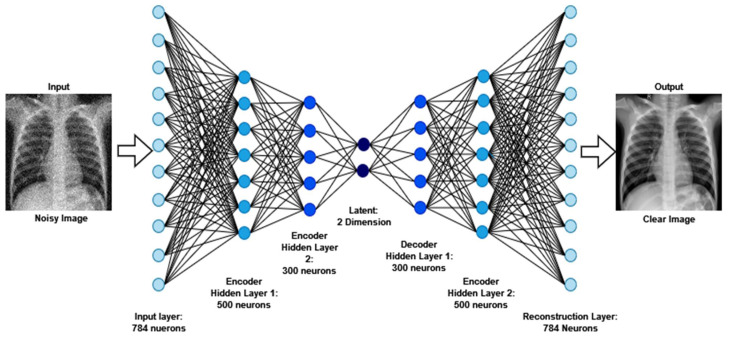
The output and architecture of the image denoising from raw noisy images.

The architecture of a convolutional neural network is characterized as having two key components: an encoder that performs feature extraction and a decoder. Essentially, the encoder scans the picture using filters and increases the depth of the image to allow for greater feature extraction, while the decoder reconstructs the same image. The results after denoising images are exhibited in Figure 3.

#### 3.1.2. Image Annotation Remove

The objective of image text erasing is to enshrine the text and replace it with a visually convincing backdrop while leaving the non-text sections untouched. The following are the difficulties associated with deleting the scene text: (1) image text removing methods should be capable of anticipating the text’s stroke-level position, which is more complicated than the bounding-box-level scene text recognition methods that have been extensively researched [54]; (2) after trying to remove words, the source text region must be loaded with a fresh visually plausible background; and (3) non-text regions should retain their original appearance. The EnsNet [55] network is used to remove text from images. It is made of three seamlessly linked modules: (1) the refine generating core; (2) the finely tuned module; and (3) the EnsNet discriminator. The network is end-to-end trainable with an extraordinarily high efficiency. 

EnsNet is made up of two fundamental components: a generator G and a discriminator D. EnsNet aims to construct a non-text picture *y* that is as real as appropriate, given a scene text image x and a ground truth z, by solving the optimization problem [56], which may be described using Equation (2).

(2) $$min_{G}max_{D}E_{x\sim p_{data\left(x\right)\;},\;\;\;z}\left[\log\left(1-D\left(x,G\left(x,z\right)\right)\right)\right]+E_{x\sim p_{data\left(x\right)\;},\;\;\;z}\left[\log D\left(x,y\right)\right]$$

Here, *G* is composed of three modules that are mutually promoted: (1) a lightweight Fully-Convolutional Networks (FCN) core, (2) lateral connections, and (3) reduced loss. 

(1) The FCN is composed of a convolutional and a deconvolutional route. The former makes use of a low-power Resnet18 network. To forecast the text/non-text score map, the 1 × 1 convolutional layer transformed with the last layer is put on top of the Resnet18’s final convolutional layer. The deconvolution route is composed of 5 deconvolutional layers, each having a kernel size of 4, a stride step size of 2, and a padding size of 1.

(2) The lateral connections often are thought to have better semantics, while the higher-level features have weaker semantics but include more specific information, such as pixel-level color, texture, and object location information [57]. As a result, they created lateral links to relate higher-level semantics to lower-level features. A transforming block and an upsampling block are included in the proposed lateral connection. The transforming block begins with a shrinking layer that uses a 1 × 1 convolution to minimize the feature dimensions. Following that, two convolutional layers of the same size (3 × 3) are stacked to provide a nonlinear transformation that not only replaces the sizable convolution layer [58] but also improves the processing efficiency. Finally, an expanding layer is utilized to reverse the shrinking step by enlarging the convolutional feature channels using a 1 × 1 convolution. The transformation block accepts as input the Residual2b to Residual5b of the Resnet18. In addition, a deconvolutional layer is employed to increase the feature map in the upsampling block [59]. The upsampled features maps are then added element-by-element to the converting block’s corresponding ones. Additionally, unlike earlier CNN-based approaches, all ReLU/LeakyReLU levels, except those in the convolution route, are replaced with ELU layers after each layer.

(3) Refined loss algorithms take into account both per-pixel reconstruction accuracy and composition, such as how well text sections blend into their surrounding context. This generative network is equipped with four functions: (i) loss of multiscale regression, (ii) loss of content, (iii) loss of texture, and (iv) loss of total variation.

In the multiscale regression loss, an input picture containing text $I_{in}$, an initial binary mask M, a generator output $I_{out}$, a ground truth image $I_{gt}$, and extracted features from several deconvolutional layers are provided to generate outputs of varying sizes. This allows for the gathering of additional contextual information at various sizes, where $I_{out\left(i\right)}$ denotes the ith deconvolutional output, $M_{i}$ and $I_{gt\left(i\right)}$ denote the segmentation masks and ground truth with the same scale as $I_{out\left(i\right)}$, and weights are given of the relevance of the text and non-text areas. The weight of the ith scale is denoted by $\lambda_{i}$. The loss function for the multiscale regression is defined in Equation (3).

(3) $${ℒ}_{m}\left(M,I_{out},I_{gt}\right)=\sum_{i=1}^{n}\lambda_{i}\left(∥M_{i}⊙\left(I_{out\left(i\right)}-I_{gt\left(i\right)}\right)∥1+\alpha ∥\left(1-M_{i}\right)⊙\left(I_{out\left(i\right)}-I_{gt\left(i\right)}\right)∥1\right)\;$$

The content loss function has been shown to be effective for recreating high-level features [60]. To further improve the speed of text erasing, also included are the content limits on the high-level characteristics, as described in Equation (4); it refers to them as content loss, where $I_{comp}$ is the output image $I_{out}$, with $I_{out}$’s non-text areas set towards the ground truth. The activation map for the nth chosen layer is denoted by $A_{n}$.

(4) $${ℒ}_{C}=\sum_{n=1}^{N-1}||(A_{n}\left(I_{out}\right)-A_{n}\left(I_{gt}\right)||1)+\sum_{n=1}^{N-1}||(A_{n}\left(I_{comp}\right)-A_{n}\left(I_{gt}\right)||1)$$

The texture loss should be included in the optimization process as well. As a result, texture degradation is introduced, ensuring that the recovered text portions match the non-text sections. The current success of neuronal style transfers [61] has prompted the loss. Before performing the L1 loss, which may be described by Equations (5) and (6), texture loss conducts the autocorrelation [62] on every high-level feature map. When ($H_{n}W_{n}$) × $C_{n}$ denotes the form of the high-level activation map ($A_{n}$), the discrepancy between the texture appearances of the text and non-text areas is penalized so that the network may collect the global style characteristics for more fairly shifting the textual areas. Again, loss terms are included for both the unprocessed result $I_{out}$ and the text-erased areas’ output $I_{comp}$.

(5) $${ℒ}_{T_{out}}=\sum_{n=1}^{N-1}||\frac{1}{C_{n}H_{n}W_{n}}({\left(A_{n}\left(I_{out}\right)\right)}^{T}\left(A_{n}\left(I_{out}\right)\right)-({\left(A_{n}\left(I_{gt}\right)\right)}^{T}\left(A_{n}\left(I_{gt}\right)\right)||1\;$$

(6) $${ℒ}_{T_{comp}}=\sum_{n=1}^{N-1}||\frac{1}{C_{n}H_{n}W_{n}}({\left(A_{n}\left(I_{comp}\right)\right)}^{T}\left(A_{n}\left(I_{comp}\right)\right)-({\left(A_{n}\left(I_{gt}\right)\right)}^{T}\left(A_{n}\left(I_{gt}\right)\right)||1$$

The loss of total variation $L_{tv}\;$ is intended to achieve global denoising, as specified in Equations (7) and (8), where i and j denote the pixel dimensions. In Equation (8), the hyper-parameters, $\lambda_{e}$, $\lambda_{i}$, and $\lambda_{t}$, determine the balance of the four losses.

(7) $${ℒ}_{tv}=\sum_{i,j}||I_{out}^{i,j}-I_{out}^{i+1,j}\left|\left|1+\right|\right|I_{out}^{i,j}-I_{out}^{i,j+1}||1$$

(8) $${ℒ}_{refined}={ℒ}_{M}+\lambda_{e}{ℒ}_{c}+\lambda_{i}{ℒ}_{T}+\lambda_{t}{ℒ}_{tv}$$

**Figure 4:**
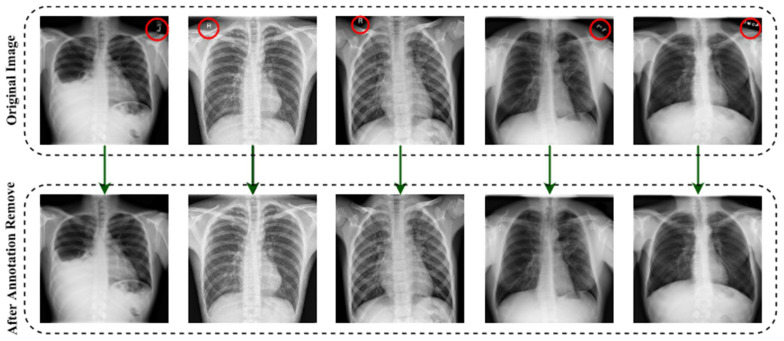
The output after deploying the image annotation removal method.

To distinguish between false and genuine photos, the initial GANs [63] differentiate the results at the entire image level. However, as the non-text portions comprise a large amount of the picture and are often genuine, it is difficult for the discriminator to concentrate on the text sections. As a result, it is natural to build a local-aware discriminator that is conscientious about the consistency between the text-erased patches and their underlying texture. The output after applying the image annotation method is (EnsNet) illustrated in Figure 4. In this figure, the annotations marked in red circles.

#### 3.1.3. Image Enhancement (CLAHE)

To enhance the contrast of an image, Contrast Limited Adaptive Histogram Equalization (CLAHE) is utilized. CLAHE is a more advanced version of Adaptive Histogram Equalization (AHE). The research in [64] examined the two approaches and stated why CLAHE is preferable to AHE. CLAHE was developed to improve the quality of the imaging of complex structures in medicine [65]. CLAHE improves the local contrast of medical imaging and its usability [66]. 

CLAHE has been shown to be effective at enhancing low-contrast pictures [67]. The CLAHE method divides the pictures into contextual sections known as tiles and generates a histogram of each one and approximates the result to a given histogram distribution parameter. The study in [68] provided a concise description of the mathematical formulas regulating CLAHE. Imagine that a picture is N×N pixels in size and each tile in the image is n×n pixels in size. The overall quantity of tiles is then calculated using Equation (9),

(9) $$T=\frac{N\times N}{n\times n}$$

The necessary histograms of the above tiles are constructed by exploiting the clip limit, $C_{L}$ as specified in Equation (10).

(10) $$C_{L}=N_{CL}\times N_{AVG}$$

Here, $N_{CL}$ stands for Normalized Contrast Limit. $N_{AVG}\;$ is the average count of pixels in the picture. The ratio of $N_{AVG}$ is determined using Equation (11).

(11) $$N_{AVG}=\frac{N_{x}\times N_{y}}{N_{g}}$$

Here, $N_{g}$, $N_{x}$, and $N_{y}$ in the equation represent the number of grayscales, the pixel size in the x dimension, and the pixel size in the y dimension, respectively, in the tile. The number of mean values of clip pixels is calculated using Equation (12).

(12) $$N_{cp}=\frac{N\sum C_{L}}{N_{g}}$$

Here, $C_{L}$ stands for clipped pixels, while $N\sum C_{L}$ stands for the overall number of $C_{L}$. Some pixels remain after the $N_{cp}$ is spread across the various gray levels. It is used to redistribute the leftover pixels, using Equation (13).

(13) $$R=\frac{N_{g}}{N_{r}}$$

where R is calculated by dividing $N_{g}$ by the image pixels to be reallocated, $N_{r}$. To reduce false borders in the image data, bi-linear interpolation is used to combine neighboring tiles. 

**Figure 5:**
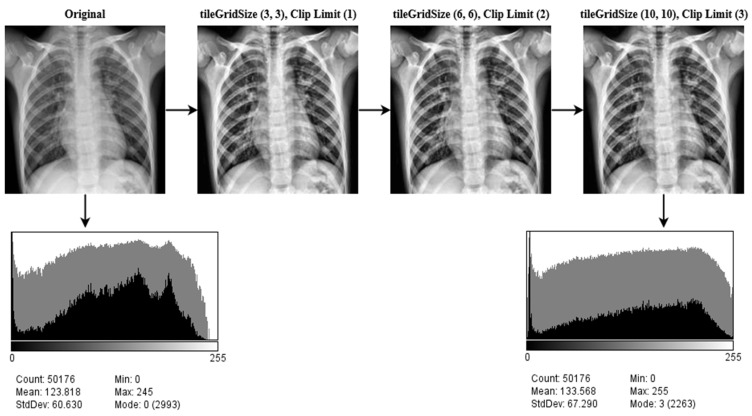
The result of CLAHE enhancement method implemented on ten classes: Control, COVID-19, Effusion, Lung Opacity, Mass, Nodule, Pulmonary Fibrosis, Pneumonia, Pneumothorax, and Tuberculosis, applying several tileGridSize (10, 10) and clip limit (3) permutations.

In order to overcome the limitations of global approaches, the CLAHE methodology focuses on increasing local contrast. The size of the tiles and the clip limit are key hyper-parameters for this approach. An erroneous selection of hyper-parameters may have a significant impact on the quality of the picture. Numerous values for these parameters are evaluated, and the ones that provide the best results are picked for tileGridSize (10, 10), the clip limit (3). An image histogram of the CLEHE and the raw pictures is created to indicate the intensity levels of the image pixels. A statistical representation of the pictures is used in the study, with an intensity range of pixel 0–255. As seen in the histogram, the contrast of a CLAHE picture is significantly superior to that of the original raw image. The output results after applying CLEHE are shown in Figure 5. 

#### 3.1.4. Filter Apply

**Figure 6:**
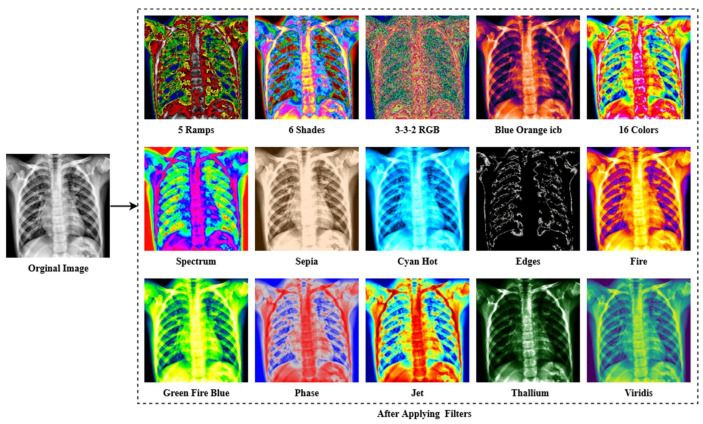
The result of different filtering algorithms includes the following: 5 Ramps, 6 Shades, 3-3-2 RGB, Blue Orange icb, 16 colors, Spectrum, Sepia, Cyan Hot, Edges, Fire, Green Fire Blue, Phase, Jet, Thallium, and Viridis were applied to the X-ray image dataset.

**Figure 7:**
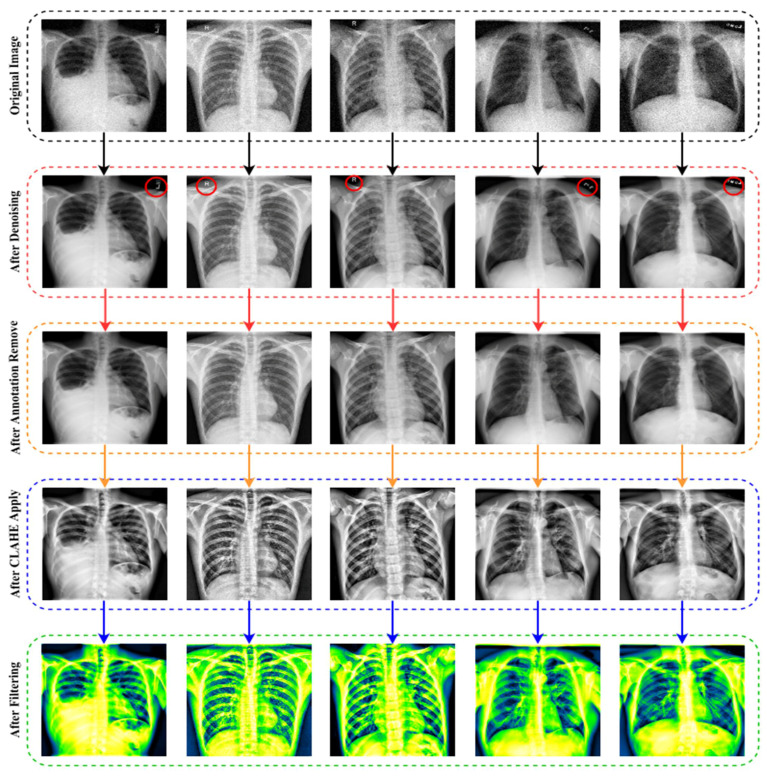
The final outcome following the use of all image preprocessing methods.

The last enhancing technique in this research is to apply a filter. The fundamental concept of filtering is that single pixels in a picture are given a new value based on the values of the adjacent pixels within a specific zone. Different filters generate their output by doing various computations on the neighborhood. This filtering technique is used to enhance the image’s properties and render it more identical to the model for training, validation and testing. As shown in Figure 6, multiple filters (5 Ramps, 3-3-2 RGB, 6 shades, 16 colors, Blue Orange icb, Spectrum, Fire, Sepia, Cyan Hot, 16 colors, Edges, Phase, Jet, Green Fire Blue, Thallium, and Viridis) are applied to the X-ray pictures, and the optimum filter with the excellent accuracy is chosen. The improved picture is given as output in Figure 7, following the application of the CLAHE procedures.

As seen in Figure 6, the lung damage is more easily recognized with Green Fire Blue (GFB) than with the other filters. However, as indicated in Section (Ablation Study of the Applied Filters) below, where some other filters have already been evaluated as well. Furthermore, the GFB filter achieves the desired result on the dataset. To feed the model with a better image and obtain a satisfactory result, we performed a variety of preprocessing approaches to the dataset, including image denoising, image annotation removes, image enhancement using CLAHE, and image filtering. Figure 7 depicts the final result after using all of the picture preparation techniques described.

##### Ablation Study of the Applied Filters 

A number of preprocessing techniques were applied to the dataset to obtain a satisfying outcome. The validation accuracy is given as ‘Val Acc’ in Table 3, whereas the test accuracy is indicated as ‘T_Acc’; validation loss is indicated as ‘Val_Loss’ and test loss is indicated as ‘T_Loss’. From Table 3, fifteen filters have been evaluated to feed the model and obtain the highest accuracy. ‘Green Fire Blue’ achieved the highest of all the filters with Val_Acc 98.03%, along with T_Acc 98.89%, Val_loss 0.11%, and T_Loss 0.09%, respectively.

### 3.2. Image Augmentation

To perform well, a deep learning algorithm requires a large number of inputs. In this study, many augmentation techniques are used to increase the enhanced data. By augmenting the training datasets with extra and unique samples, data augmentation may help to enhance the performance and results of the machine learning algorithms. If the datasets used to train the algorithm are suitably large and diverse, the method works better and is highly accurate. Through the utilization of image augmentation techniques, the accuracy of the findings is improved. Additionally, data augmentation techniques are an excellent tool for diversifying the datasets. Generally, data augmentation techniques have been used to expand the volume of the training sets in order to provide more relevant training data to large-capacity learners. However, there is a new tendency developing among the research community on deep learning in which samples are enhanced through the test data augmentation method [69,70,71,72]. Test data augmentation can help increase the robustness of a trained model [73,74,75]. Test data augmentation may be utilized to enhance deep network prediction performance and open up new intriguing possibilities for medical image interpretation [76,77,78]. The most often utilized methods for augmenting data include rotating, mirroring, flipping, zooming, and cropping. 

**Figure 8:**
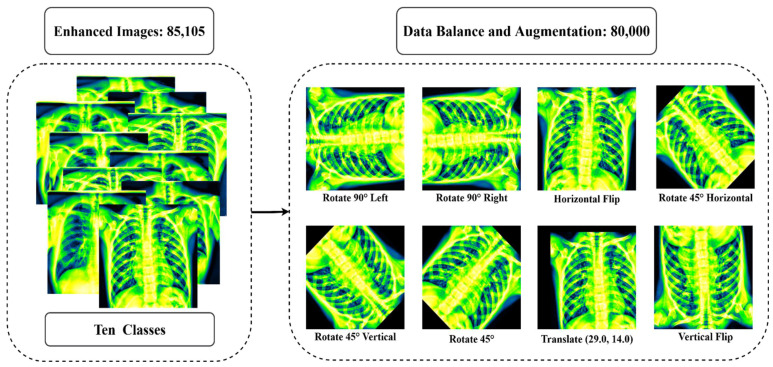
The outcome after balancing all class and using eight augmentation procedures: Rotate 90° left, Rotate 90° right, Horizontal flip, Rotate 45° Horizontal, Rotate 45° Vertical, Rotate 45°, Translate, Vertical flip.

In this study, the dataset is collected and merged from various sources as the amount of data in each class is not balanced (see Table 2). To balance the dataset, oversampling and undersampling methods are utilized. Firstly, a random undersampling strategy is applied to balance the class (control, COVID-19, Effusion, and Pneumonia) with excessive data. This method randomly deletes data from the majority classes and the number of data is reduced to 8000 data per class. Then, the oversampling (data augmentation) strategy is used to balance the class (Lung Opacity, Mass, Nodule, Pulmonary Fibrosis, Pneumothorax, and Tuberculosis) with insufficient data. Eight augmentation strategies are used in this study: Rotate 90° left, Rotate 90° right, Horizontal flip, Rotate 45° Horizontal, Rotate 45° Vertical, Rotate 45°, Translate and Vertical flip on pre-processed data. Table 4 summarizes all of the augmentation parameters. After augmentation, the dataset was increased to comprise 80,000 images (Control: 8000, COVID-19: 8000, Effusion: 8000, Lung Opacity: 8000, Mass: 8000, Nodule: 8000, Pulmonary Fibrosis: 8000, Pneumonia: 8000, Pneumothorax: 8000, Tuberculosis: 8000). Figure 8 shows the outcome of the data augmentation.

Before training begins, the dataset must be divided. The X-ray images were divided into three groups according to the 60:20:20 split across the training, validation, and test sets. After dividing the 80,000 X-ray images of the dataset into three subsets, the training set comprises 48,000 X-ray images, the validation set includes 16,000 X-ray images, and the testing set contains 16,000 X-ray images. Table 5 summarizes the final description of the dataset after all the pre-processing steps.

## 4. Proposed Model

One of the primary objectives of this study is to produce adequate classification results utilizing the transfer learning models on the merged dataset. Eight pre-trained models, including AlexNet, GoogLeNet, InceptionV3, ResNet50, MobileNetV2, EfficientNetB7, DenseNet121, and VGG16, are evaluated to determine the most efficient transfer learning method for the classification assignment. By adding several layers to the VGG16 model, a customized fine-tuned transfer learning method was developed and deployed to achieve the highest accuracy among all the other pre-trained models. 

### 4.1. Transfer Learning Models 

#### 4.1.1. AlexNet

AlexNet is a popular convolutional neural network model. Max pooling, convolutions and dense layers are among the key building components. The model is fitted using grouping convolutions over two GPUs. Alexnet is made of eight layers within each set of learnable parameters. The model consists of five convolutional layers that combine fully connected and max pooling layers, along with two normalizing layers and one softmax layer. Each layer is built of a convolutional layer and an ReLU-based nonlinear activation function. Max pooling is accomplished by the usage of pooling layers. The input size 224 × 224 × 3 pixel is fixed due to the perceived layers. An RGB image is created whenever a gray picture is utilized as an input, and this is done by multiplying the single channel. The batch size is 128, and the model includes 60 million parameters.

#### 4.1.2. GoogLeNet

GoogLeNet is a convolutional neural network architecture modeled after the inception architecture. Googlenet attempts to increase computing efficiency by using an iterative algorithm as the fundamental layer. Subsequent layers are added on top of each other and perform parallel filtering on the input from the preceding layer. It makes use of inception modules, which let the network choose from a range of convolutional filter sizes inside each block. With a stride of two, these modules may be layered on top of each other to create an inception network that reduces the grid’s resolution. In comparison to the inception design, GoogLeNet is a deep neural model with 22 layers and fewer parameters. The network has been pre-trained to accept pictures with a quality of 224 × 224 pixel. The design is composed of three layers: Activation, AveragePooling2D, and Dense. Global average pooling was used in GoogLeNet rather than a fully connected layer. 

#### 4.1.3. InceptionV3

InceptionV3 reduces the amount of computer power required by updating prior inception designs. Factorized convolutions, normalization, down sampling, and parallelized calculations are only a few of the techniques used to minimize the computational cost. The training time of the InceptionV3 model is decreased by replacing smaller convolutions with larger convolutions. Numerous optimization techniques for removing restrictions and increasing the flexibility of an InceptionV3 model have been developed. Data preprocessing is a vital element as it has a considerable influence on the maximum accuracy of the model during training. At a minimum, pictures should be classified and scaled to correspond to the model. Pictures must be 299 × 299 × 3 pixel in size for Inception. Average pooling, convolutions, max pooling, dropouts, concatenations, and fully-connected layers are used to create the model. The model makes extensive use of batch normalization on activation inputs. The loss is calculated using SoftMax.

#### 4.1.4. ResNet50

ResNet50 is a 50-layer neural network (CNN) composed of 48 fully connected layers, a max pool layer, and an average pool layer. It is capable of doing floating-point calculations upwards of 3.8 × 10^9^. The ResNet50 architecture makes use of a mixture of convolutional filters of varying sizes to overcome the decay issue inherent in CNN models and to shorten the time for training connected with the deep structure. ResNets contain few filters and so perform more quickly. The performance of the 34-layer ResNet is 3.6 billion FLOPS, compared with 1.8 billion FLOPS for the shorter 18-layer ResNets. Around 23 million variables may be used to train this design. The network may accept an input image with dimensions that are multiples of 32 in width, height, and channel width. Each ResNet architecture employs a 7 × 7 kernel size for early convolution and a 3 × 3 kernel size for max pooling, respectively. Each two-layer block in the 34-layer net is substituted with this three-layer bottleneck block, culminating in a 50-layer ResNet.

#### 4.1.5. MobileNetV2

In MobileNetV2, a unique module which includes an inverting residual structure is added. MobileNetV2 enables the identification and segmentation of objects at the cutting-edge of technology. The design of MobileNetV2 starts with a fully connected convolution layer composed of 32 filters and 19 residual bottlenecks layers. MobileNetV2 is a convolutional neural network with 53 layers. It is a pre-trained classifier that has been trained on over a million photos from ImageNet. The network typically needs 3 billion multiply-add processes and makes use of 3.4 million variables. The model was pre-trained on photos classified into 100 distinct item categories. As a result, the network has retrieved a large number of characteristics from a diverse group of photos. The topology of MobileNetV2 starts with such fully convolutional layers comprised of 32 filters and 19 residual bottlenecks. It is divided into two different blocks, each with three layers. Both blocks start and conclude with a 1 × 1 convolution layer containing 32 filters, whereas the second level layer is indeed a depth-wise fully connected layer. Throughout various levels of the architecture, the ReLU is employed. The difference between the two blocks lies within the stride size, having blocks 1 stride length of 1, while block two has a stride length of 2.

#### 4.1.6. EfficientNetB7

A new benchmark network was developed utilizing the AutoML MNAS architecture to boost performance, which increases the overall effectiveness and precision (FLOPS). In that it uses mobile inversion bottleneck convolution (MBConv), the resulting architecture is similar to MobileNetV2 and MnasNet; however, it is somewhat larger due to an increased FLOP limit. FLOPS are used to evaluate how well the algorithm performs in terms of correctness and efficiency. The MBConv is used in this architecture. The researchers subsequently increased the size of this first network in order to create the EfficientNets series of deep learning models. EfficientNets are a series of models derived from the original EfficientNet. The pre-trained weights are not included in EfficientNetB7. EfficientNet-B7, the most recent version of EfficientNet, offers the best accuracy of all of the versions and requires the fewest parameters. 

#### 4.1.7. DenseNet121

In a feed-forward CNN, each convolutional layer saves the first, obtains the output from the preceding convolutional layer and generates outputs from extracted features which are then carried onto the next convolutional layer. As a result, there seem to be ‘L’ direct connections between ‘L’ layers, one between every layer and the following layer. Nevertheless, as the number of hidden layers inside the CNN increases, the ‘vanishing gradient’ issue becomes apparent. This implies that when the route from the feed to the production layers becomes longer, some information may ‘vanish’ or get lost, impairing the network’s capacity to train properly. DenseNets address this issue by altering the typical CNN design and streamlining the layer connection pattern. Each layer in a DenseNet design is directly linked to every other layer, resulting in the term Densely Linked Convolutional Network. Between ‘*L*’-levels, there are L(1+1)/2 direct links. No average is used; instead, the extracted features from previous levels are combined and used as inputs in the subsequent layers. By rejecting duplicate feature maps, DenseNets are able to reuse features as they need less parameters than a similar traditional CNN. In dense blocks, the quantity of filters fluctuates; however, the size of the feature maps does not. These intermediate layers, known as transition layers, are accountable for down-sampling the picture utilizing batch normalization, 1 × 1 convolution, and 2 × 2 pooling layers.

#### 4.1.8. VGG16

The VGG16 DCNN model was developed by Simonyan and Zisserman [79]. Increasing the depth of the VGG model may help the kernels in learning more complex properties. A pre-trained VGG16 network beat the fully trained networks by a large margin in experiments on the effectiveness of transfer learning [80]. This network is trained on images from the ImageNet collection totaling over a million. The network is composed of 16 layers and is capable of recognizing images belonging to a variety of categories. The conv1 layer is fed 224 × 224 RGB pictures of standard size. The picture is convoluted using a succession of filters tuned to create the lowest possible receptive field: 3 × 3. Furthermore, one of the pairings utilizes a 1 × 1 convolutional filter, which changes the input channels linearly. The stride of convolution is set to one pixel, and the spatial padding of the convolution layers’ input is adjusted to retain spatial resolution after convolution. Padding, for example, is 1 pixel for 3 × 3 convolutional layers. Following the convolutional layers, five max-pooling layers are employed to achieve spatial pooling. Stride 2 is being utilized to perform max-pooling operations across 2 × 2 pixel frames. Three convolutional layers are placed on top of one another. The first two contain 4096 channels, respectively, whereas the third does ILSVRC classification on a 1000-way basis. Finally, there is a softmax layer. In all networks, the structure of the entire linked levels is the same.

#### 4.1.9. LungNet22

**Figure 9:**
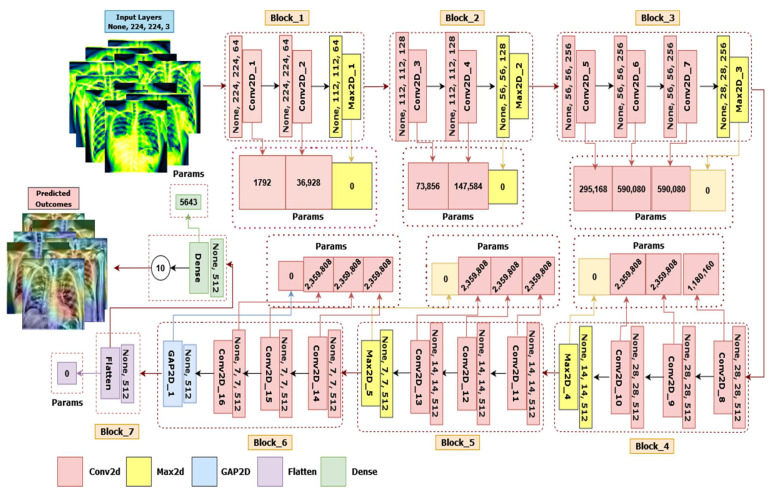
The architecture of LungNet22, a fine-tuned VGG16 network.

In terms of classification performance, the fine-tuned VGG16 architecture named LungNet22 surpasses the other eight model architectures discussed above. As a consequence, the LungNet22 model is proposed and validated using our merged dataset, which is constructed on VGG16 architecture. Furthermore, ablation research is being carried out to improve the architecture’s endurance concerning lung disease classification. The architecture of the model is illustrated in Figure 9.

The dataset contains 80,000 images classified into ten categories: Control, COVID-19, Effusion, Lung Opacity, Mass, Nodule, Pulmonary Fibrosis, Pneumonia, Pneumothorax and Tuberculosis. The dataset is divided into 60% for training, 20% for validation, and 20% for testing. All tests were conducted on an AMD Ryzen 7 3800X, with base clock 3.90–4.50 GHz CPU with eight (32 MB L3 cache, storage speed = 3200 MHz) cores and sixteen threads, 64 GB of RAM, and the Anaconda 3 (Jupyter Notebook) platform. It is integrated with an AMD Radeon RX 580 series GPU. The Python programming language is used to obtain the findings.

LungNet22 is formed by connecting two blocks after VGG16’s fifth block. The sixth block has three convolutional layers and one GlobalAveragePooling2D layer, while the seventh block contains a flatten layer connected to a dense layer. As the input layer of the network default required a size of 224 × 224 × 3 pixel for the picture, it is an RGB image. The first block in this proposed architecture has two convolutional layers with 64 channels of 3 × 3 kernel size, an ReLU activation function, and the same padding preceded by a Maxpooling2D layer of 2 × 2 stride and a pool size of 2 × 2. Similar to the first block, the second block has two convolution layers with 128 channels of kernel size 3 × 3, an ReLU activation function and the same padding preceded by a Maxpooling2D layer with a stride of 2 × 2 and a pool size of 2 × 2. In blocks 3, 4, and 5 are three convolutional layers followed by one Maxpooling2D layer. These three convolutional layers have channel lengths of 256, 512, and 512, sequentially, with the same kernel size of 3 × 3, the same padding, and an ReLU activation function. All of these blocks with a Maxpooling2D layer also performed as a last layer with a stride and a pool size of 2 × 2. Each Maxpooling2D layer compresses the input image to twice its initial value size. 

Block six has three convolutional layers preceded by a GlobalAveragePooling2D layer. The convolutional layers have the same kernel size of 3 × 3, the same padding, and an ReLU activation function with 512 channels. A flatten layer has been also added in block 7, connected to dense a layer which outputs for the 10 classes. The Softmax activation and Adam optimizer were applied to the final layer. A speed of learning of 0.000001 is used throughout the method. Finally, we assessed the accuracy, recall, precision, and f1-score.

### 4.2. Ablation Study of the Proposed Model (LungNet22)

Four experiments were conducted as an ablation study by modifying various aspects of the proposed LungNet22 architecture depending upon the fine-tuned VGG16 model. By modifying different components, it is possible to develop a more resilient architecture with increased classification accuracy. The following aspects were subjected to an ablation study: Global AveragePooling2D (GAP2D), Flatten layer, Loss function, Optimizer, and Learning rate.

#### 4.2.1. Ablation Study 1: Changing GlobalAveragePooling2D Layer

In this study, the validation loss is indicated by the abbreviation ‘Val_Loss’; the validation accuracy is represented by the abbreviation ‘Val_Acc’; the test loss is defined by the abbreviation ‘T_Loss’; and the test accuracy is stated by the abbreviation ‘T_Acc’. Table 6 shows that for the GlobalAveragePooling2D layer, the LungNet22 has the greatest results, with a Val_Acc of 97.03% and a T_Acc of 98.89%. However, the accuracy of the GlobalMaxPooling2D, AveragePooling2D, and Maxpooling2D layers decreases slightly, with Val_Acc values of 95.73%, 94.92%, and 95.77%, and T_Acc values of 96.12%, 95.30%, and 96.19%, consecutively.

#### 4.2.2. Ablation Study 2: Changing the Flatten layer

The validation accuracy is labeled ‘Val_Acc’; the validation loss is labeled ‘Val_Loss’; the test accuracy is labeled ‘T_Acc’; and the test loss is labeled ‘T_Loss’. GlobalAveragePooling2D, GlobalMaxPooling2D, AveragePooling2D, and Maxpooling2D change the flatten layer to see how it affects the model effectiveness. As shown in Table 7, GlobalAveragePooling2D’s accuracy drops marginally with the T_Acc of 96.94%. The network achieves the lowest results for GlobalMaxPooling2D, AveragePooling2D, and Maxpooling2D, respectively, with T_Acc values of 91.12%, 93.21%, and 95.16%.

#### 4.2.3. Ablation Study 3: Changing Loss Functions

To identify the ideal loss function for the new proposal, a variety of loss functions were explored, namely Cosine Similarity, Categorical Crossentropy, and Mean Squared Error. Table 8 illustrates the model’s performance using the specified loss functions. When fitted with Categorical Crossentropy, the model achieved the maximum test accuracy of 98.89 percent. The loss functions for Cosine Similarity and Mean Squared Error resulted in a modest decrease in test accuracy of 96.16% and 94.20%, respectively. To attain the greatest classification performance, the Categorical Crossentropy loss function was selected for further exploration.

#### 4.2.4. Ablation Study 4: Changing Optimizer and Learning Rate 

The validation loss is labeled ‘Val_Loss’; the validation accuracy is labeled ‘Val_Acc’; the test loss is labeled ‘T_Loss’; and the test accuracy is labeled ‘T_Acc’. As shown in Table 9, the optimizer ‘Adam’ with a learning rate of 0.000001 increased the model performance with the T_Acc of 98.89% and the Val_Acc of 97.03%. The ‘Adam’ optimizer still had the least T_Loss of 0.09, with a learning rate of 0.000001. Other optimizers, such as SGD, RMSprop, and Nadam, scored very efficiently in our model, with T Acc values above 90%.

## 5. Results

The study used a total of 80,000 frontal chest X-ray image data that were divided into 10 classes, namely Control, COVID-19, Effusion, Lung Opacity, Mass, Nodule, Pulmonary Fibrosis, Pneumonia, Pneumothorax, and Tuberculosis. Each class contains 8000 image data. These data are preprocessed before CNN models are applied to them. All the images in the balanced dataset are resized to 224 × 224 pixel to ensure the same scale. The dataset is divided into training, validation, and test sets. The training set is comprised of 60% or 48,000 data. The validation and test sets are both made up of 20% data from that dataset, i.e., 16,000 data in each set. Initially, eight pre-trained CNN models are trained, validated and tested. From them, the model with most accurate performance is selected for fine-tuning to create a novel LungNet CNN model to classify lung disease. 

**Figure 10:**
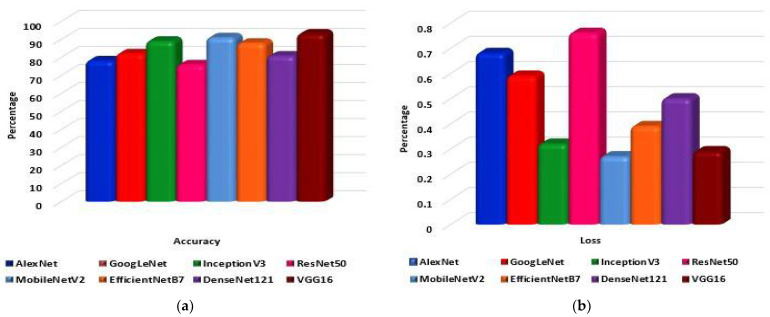
The eight pre-trained models: (**a**) Accuracy and (**b**) Loss comparative graph to classify lung disease.

To begin with, eight pre-trained CNN models are trained and tested on the dataset. The models are AlexNet, GoogLeNet, InceptionV3, ResNet50, MobileNetV2, EfficientNetB7, DenseNet121, and VGG16. Each model performance has 300 epochs to classify the 10 classes. As shown in Figure 10a, VGG16 classifies the data with 92.95% accuracy, followed by MobileNetV2 with 90.90% accuracy. InceptionV3 classifies data with 88.98% accuracy. Subsequently, EfficientNetB7, GoogLeNet, DenseNet121, AlexNet, and ResNet50 can classify the data with 87.85%, 81.98%, 80.77%, 78.09%, and 75.85% accuracy, respectively. From Figure 10b, it is observed that the highest loss is shown by GoogLeNet and the lowest loss by MobileNetV2, with 0.61% and 0.29%, respectively. The model VGG16 has a loss of 0.36%. 

It is observed that the VGG16 has the highest classification accuracy among the implemented pre-trained models. Fine-tuning the said model, the proposed LungNet22 is established. The proposed model performs 300 epochs on the dataset. To optimize loss, optimizer ‘Adam’ is employed. The learning rate of the model is 0.000001. The model is fed with pre-processed image of 224 × 224 pixels to achieve optimal accuracy. 

**Figure 11:**
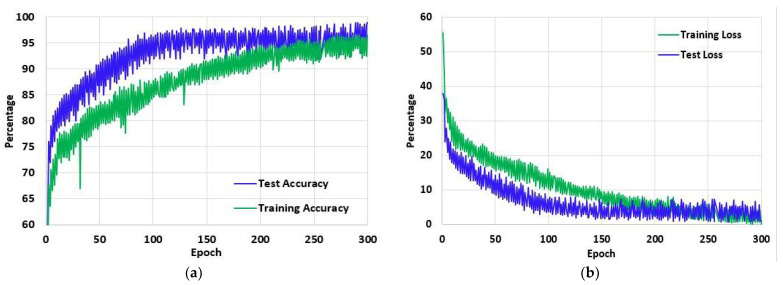
The (**a**) Accuracy and (**b**) Loss of the proposed model LungNet22 for 300 epochs using ‘Adam’ optimizer with learning rate 0.000001.

Figure 11a shows the training and test accuracy of the proposed model over the 300 epochs. It can be observed that the accuracy curve overall has increased significantly and that with the increasing epoch denotation the performance of the model improves considerably. In the first epoch, the training accuracy of the model is 53.08% and over the epochs increased to 96.47% in the last epoch. Similarly, the test accuracy of the first epoch is 44.6%, which increased to 98.89% in the last epoch. On the other hand, in Figure 11b the loss curve of the model is presented. Unlike the accuracy curve, the loss value of the model decreased with each epoch, denoting that the performance of the model increased. Initially, the training loss was 55.56%, and the test loss was 44.6%. However, on the final epoch, the training and test loss reduced significantly to 0.15% and 0.09%, respectively. 

**Figure 12:**
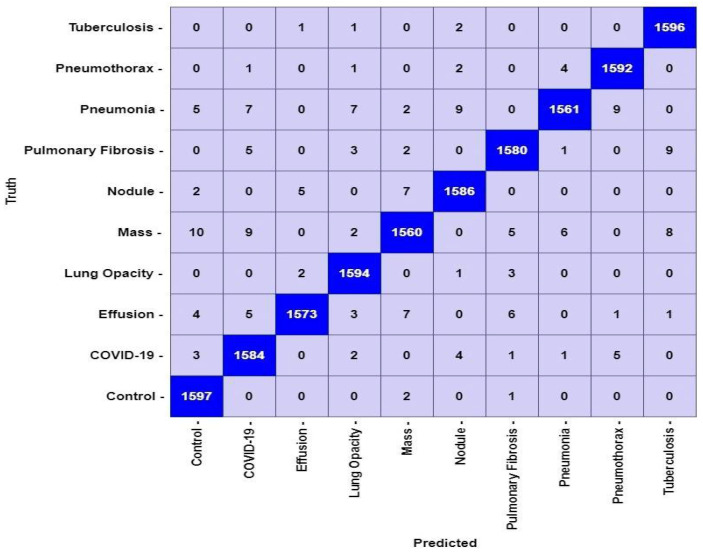
Confusion matrix for LungNet22 model.

The proposed LungNet22 can successfully classify lung disease with high accuracy. The test set contains a total of 16,000 image data. Each of the 10 classes contains 1600 data. In Figure 12, the confusion matrix of the model is illustrated. According to the confusion matrix, the model can classify a total of 15,823 data correctly. Of these, 1597 belong to the Control class and 1584 belong to the COVID-19 class. The model can accurately classify 1573, 1594, and 1560 data from the Effusion, Lung Opacity, and Mass classes, respectively. From the classes of Nodule, Pulmonary Fibrosis, Pneumonia, Pneumothorax, and Tuberculosis, the model can correctly identify 1586, 1580, 1561, 1592, and 1596 data, respectively. A total of 177 data are misclassified by the proposed LungNet22 model.

From the confusion matrix in Figure 12, the performance of Lungnet22 is calculated. To determine the performance, measures such as accuracy, precision, recall, specificity, and f1-score are considered where accuracy refers to the percentage of corrected classified data. Precision refers to the percentage of positive classified data that was relevant. Recall or sensitivity indicates the percentage of correctly predicted positive data from all the positive data. Similarly, Specificity denotes the percentage of correctly predicted negative data from all the negative data. The F1-score expresses the balance between the precision and the recall values. The mathematical expression of the measures is shown in Equations (14)–(18).

(14) $$Accuracy=\frac{TP+TN}{TP+TN+FP+FN}$$

(15) $$Precision=\frac{TP}{TP+FP}$$

(16) $$Sensitivity/Recall=\frac{TP}{TP+FN}$$

(17) $$\mathrm{Specificity}=\frac{TN}{TN+FP}$$

(18) $$F1-Score=2\left(\frac{Precision\times Recall}{Precision+Recall}\right)$$

Here, TP or true positive is the number of correctly identified positive data in a class. TN or true negative refers to the number of correctly identified negative data. FP, also known as false positive, is the number of negative data identified as positive. Similarly, FN or false negative is the number of positive data identified as positive. 

**Figure 13:**
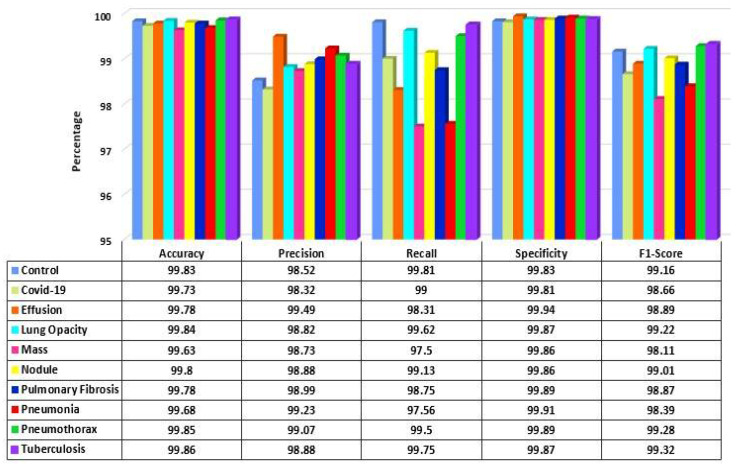
Performance score of LungNet22 network.

The performance of the proposed model is analyzed for each class of the dataset. In Figure 13, the performance measures are shown. It is observed that the model performance is high for each class. The accuracy and specificity across all the classes remained considerably consistent at over 99.60% for both. The lowest precision of the model is observed for the COVID-19 class at 98.32%, and the highest precision is for the Effusion class at 99.49%. Similarly, the Mass class shows the lowest recall value at 97.50%, and the Control class has the highest value at 99.81%. Finally, the highest F1-score is achieved in the Tuberculosis class, and the lowest is in Mass class at 99.32% and 98.11%, respectively.

**Figure 14:**
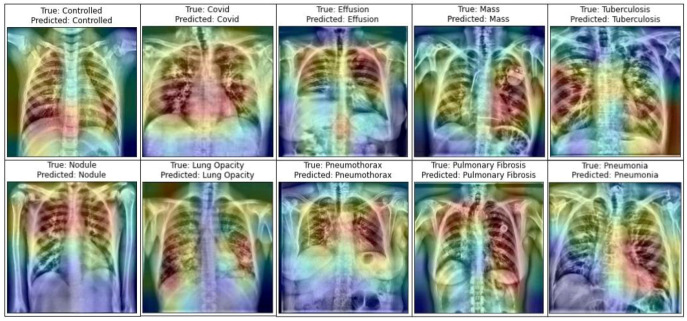
Representation of infected area of lung in X-ray images utilizing Grad-CAM on the LungNet22 model.

A number of research attempts have been performed to improve the practicability and explicability of deep learning. Additionally, it is vital to enhance the understandability of deep neural models in various deep learning applications for medical imaging. Selvaraju et al. [81] demonstrated the functioning of deep learning using a method entitled Gradient Weighted Class Activation Mapping (Grad-CAM). Grad-CAM visualizes any densely linked neural network. This enables the model’s extra information to be determined while performing classification or prediction operations. The input is a standard X-ray image, and the proposed framework is employed as a detection approach. Grad-CAM is performed to the final convolution layer just after the proposed model predicts the label. Figure 14 depicts the heatmaps on the X-ray pictures that are visualized using the proposed methodology.

**Figure 15:**
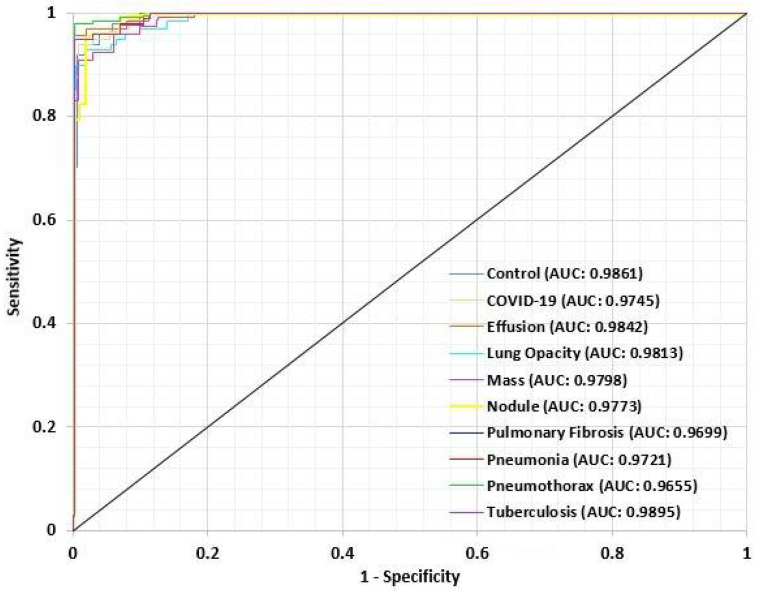
ROC curve for each class of LungsNet22.

For performance evaluation of the proposed LungNet22, an ROC (Receiver Operating Characteristics) curve is constructed for each class. The “one class versus rest” method is applied for the construction of the ROC curve for a multiclass classification task. An ROC curve is essentially a Sensitivity vs. 1-Specificity curve, where the values range between 0 to 1, with 1 indicating the best performance and 0 indicating an unsatisfactory performance. In Figure 15, the ROC curve of each class is presented. From the ROC curve, the AUC (Area Under Curve) value is computed. The AUC value of each class is recorded. The AUC value of the Control class is 0.9861. For the COVID-19, Effusion, Lung Opacity, Mass, Nodule, Pulmonary Fibrosis, Pneumonia, Pneumothorax, and Tuberculosis classes, the AUC value is 0.9745, 0.9842, 0.9813, 0.9798, 0.9773, 0.9699, 0.9721, 0.9655, and 0.9895 respectively. The AUC values overall are close to 1 for the 10 classes in the dataset, indicating that the model performs very efficiently in the classification of the lung images in each class.

## 6. Discussion

The research experiments evaluated the classification and detection of lung diseases, as well as the diagnosis of various lung diseases (COVID-19, Effusion, Lung Opacity, Mass, Nodule, Pulmonary Fibrosis, Pneumonia, Pneumothorax, and Tuberculosis) using eight pre-trained deep CNN models. On the base of a fine-tuned VGG16 model, the LungNet22 model is presented. To determine the best model, several training/validation ratios were utilized. Aside from accuracy and AUC, the most often utilized performance measures for lung disease detection are recall, specificity, and the F1 score, which have all been used to perform an overall study on the predictive accuracy and durability of the models. On our merged X-ray dataset, the proposed LungNet22 model obtained the highest accuracy of 98.89%, the highest specificity of 98.893%, the highest F1 score of 0.988, and the highest recall of 98.893%. Not only do our findings demonstrate a considerable increase in AUC, but they also give a more comprehensive estimate measure of accuracy, F1 score, specificity, and recall.

## 7. Conclusions

Lung illnesses are widespread worldwide. Lung Opacity, COVID-19, Effusion, Mass, Pulmonary Fibrosis, Nodule, Pneumothorax, Pneumonia, and Tuberculosis are a few examples of these. It is important to diagnose lung illness quickly. For this objective, several image processing and artificial intelligence models have been formed. The purpose of this work is to propose a fine-tuned model called LungNet22 for classifying lung diseases such as COVID-19, Lung Opacity, Mass, Effusion, Pulmonary Fibrosis, Pneumonia, Nodule, Pneumothorax, and Tuberculosis along with the Control class. The method of image annotation removal was utilized to erase the annotations from the images. Additionally, image enhancement methods were used to increase the quality of the X-ray images. The pre-processed image dataset was balanced and augmented to 80,000 images using eight augmentation techniques to increase the volume and balance of each X-ray dataset class. Eight pretrained classifiers and a fine-tuned model were evaluated to ensure the best level of accuracy. The structure of LungNet22 is based on the VGG16 model, but with new layers and hyper-parameters. The hypothesized network was conducted in an ablation study to determine how well it performed under different hyper-parameter conditions. LungNet22 scored best while using the Adam optimizer and a learning rate of 0.000001, with 96.47% training accuracy, 97.03% validation accuracy, and 98.89% testing accuracy. While performing recognition or prediction operations, the Grad-CAM was used to help in determining extra information about our proposed model LungNet22. The model with image processing, fine-tuning, and the ablation experiment has a high rate of accurate identification. The outcomes of this research demonstrate that image processing and enhancing and balancing techniques may help improve the model’s performance. As a future study, our model could be used in the categorization of other diseases using CT-scan pictures, or a hybrid model could be presented to classify the many types of lung illnesses. Cross-validation may be used to validate the assessment result and to provide mean accuracy and standard deviation on the training set as well as accuracy on the test set. 

In the supplementary materials there is an instruction video [S1] showing how to run the code and a bit on the original dataset (sample dataset is provided in the supplementary material file S1, sample dataset is provided in the supplementary material file S2).

## Figures and Tables

**Table 1:** A summary of related studies on lung disease classification.

Reference	Year	Classes	Disease	Data Type	Model	Performance Accuracy
[37]	2021	4	Normal, COVID-19, Pneumonia, Lung cancer	X-ray, CT	VGG19-CNN	98.05%
					ResNet152V2	95.31%
					ResNet152V2 + GRU	96.09%
					ResNet152V2 + Bi-GRU	93.36%
[38]	2020	15	No finding, Infiltration, Mass, Effusion, Atelectasis, Nodule, Pneumothorax, Consolidation, Plural thickening, Hernia, Cardiomegaly, Emphysema, Edema, Fibrosis, Pneumonia	X-ray	Vanilla Gray	67.8%
					Vanilla RGB	69%
					Hybrid CNN VGG	69.5%
					VDSNet	73%
					Modified CapsNet	63.8%
					Basic CapsNet	60.5%
[39]	2021	3	Normal, Pneumonia, COVID-19	X-ray	CNN + HOG	92.95%
[40]	2020	10	Infiltration, Mass, Effusion, Atelectasis, Nodule, Pneumothorax, Consolidation, Emphysema, Edema, Fibrosis	X-ray	UCMobN	85.62%
[41]	2021	2	Pleural effusion, Normal	X-ray	CNN	95%
[42]	2021	3	Normal, COVID-19, SARS	X-ray	DeTrac + AlexNet	95.66%
					DeTrac + VGG19	97.53%
					DeTrac + ResNet	95.66%
					DeTrac + GoogleNet	94.71%
					DeTrac + SqueezNet	94.90%
[43]	2021	14	Atelectasis, Cardiomegaly, Consolidation, Edema, Mass, Effusion, Emphysema, Nodule, Pneumothorax, Fibrosis, Infiltration, Plural thickening, Pneumonia, Normal	X-ray	DenseNet121	40.43%
					InceptionResNetV2	41.02%
					ResNet152V2	37.60%
[44]	2021	2	COVID-19, Not-COVID-19	X-ray	Mask R-CNN	96.98%
[45]	2021	3	No finding, COVID-19, Pneumonia	X-ray	CNN	87%
[46]	2020	2	Normal, Pneumonia	X-ray	VGG16	96.81%
					VGG19	96.58%
					NasNetMobile	83.37%
					ResNet152V2	96.35%
					InceptionResNetV2	94.87%
[47]	2021	2	Normal, COVID-19	X-ray	INceptionV3	90.43%
					ResNet50V2	91.11%
					DenseNet201	91.11%
					Combined Proposed Model	91.62%
[48]	2021	4	COVID-19 (Mild), COVID-19 (Moderate), COVID-19 (Severe), COVID-19 (Critical)	X-ray	Novel CNN	95.52%
[49]	2020	2	Normal, COVID-19	X-ray	DNN	83.4%
					CNN	93.2%
[50]	2021	2	Normal, COVID-19	X-ray	ResNet50 + SVM	94.7%
					Fine-Tune ResNet50	92.6%
					End-to-End training of CNN	91.6%
[51]	2022	4	Nodules, Atelectasis, Infection, Normal	X-ray	MARnet	83.3%

**Table 2:** The raw dataset’s description.

No.	Disease	Data	Reference
1.	Control	13,672	[21,22,23,26,27,28,31]
2.	COVID-19	15,660	[21,22,24,25,28,31]
3.	Effusion	13,501	[30,32,33,34]
4.	Lung Opacity	7179	[31,35]
5.	Mass	5603	[30,33]
6.	Nodule	6201	[30,33]
7.	Pulmonary Fibrosis	3357	[28,30,33]
8.	Pneumonia	9878	[21,22,23,28,31]
9.	Pneumothorax	6870	[29,30,33]
10.	Tuberculosis	3184	[24,26,27,28,36]

**Table 3:** Analyses of performances of various filters.

Filters	Val_Acc (%)	Val_Loss (%)	T_Acc (%)	T_Loss (%)	Finding
5 Ramps	63.88	0.89	64.32	0.87	Accuracy Dropped
6 Shades	62.71	0.94	63.45	0.90	Accuracy Dropped
3-3-2 RGB	65.33	0.86	66.01	0.83	Accuracy Dropped
Blue Orange icb	72.59	0.72	73.77	0.68	Accuracy Dropped
16 colors	82.63	0.61	83.09	0.59	Accuracy Dropped
Spectrum	81.19	0.65	81.80	0.61	Accuracy Dropped
Sepia	85.40	0.49	86.90	0.39	Accuracy Dropped
Cyan Hot	85.67	0.45	86.11	0.41	Accuracy Dropped
Edges	83.55	0.57	83.99	0.53	Accuracy Dropped
Fire	87.92	0.37	87.47	0.40	Accuracy Dropped
Green Fire Blue	97.03	0.11	98.89	0.09	Identical Accuracy
Phase	88.76	0.25	88.23	0.29	Accuracy Dropped
Jet	83.52	0.58	83.85	0.54	Accuracy Dropped
Thallium	88.19	0.28	88.99	0.22	Accuracy Dropped
Viridis	95.10	0.19	95.50	0.16	Accuracy Dropped

**Table 4:** Value of data augmentation variables.

Augmentation Parameters	Value
Rotate left	90°
Rotate right	90°
Horizontal flip	True
Rotate	45°
Rotate Horizontal	45°
Rotate Vertical	45°
Vertical flip	True
Translate	x, y (29.0, 14.0)

**Table 5:** The final dataset after pre-processing.

Features	Properties
Number of Images	80,000
Number of Classes	10
Enhancement and Color Grading	CLAHE, Green Fire Blue
Number of Augmentation Techniques	8
Control	8000
COVID-19	8000
Effusion	8000
Lung Opacity	8000
Mass	8000
Nodule	8000
Pulmonary Fibrosis	8000
Pneumonia	8000
Pneumothorax	8000
Tuberculosis	8000

**Table 6:** Changing the GlobalAveragePooling2D (GAP2D) layer to evaluate the ablation study.

Case Study	Layer Name	Val_Acc (%)	Val_Loss (%)	T_Acc (%)	T_Loss (%)	Finding
1	GlobalAveragePooling2D	97.03	0.11	98.89	0.09	Identical Performance
	GlobalMaxPooling2D	95.73	0.27	96.12	0.23	Accuracy Dropped
	AveragePooling2D	94.92	0.35	95.30	0.27	Accuracy Dropped
	MaxPooling2D	95.77	0.21	96.19	0.25	Accuracy Dropped

**Table 7:** Changing the Flatten layer to evaluate the ablation study.

Case Study	Layer Name	Val_Acc (%)	Val_Loss (%)	T_Acc (%)	T_Loss (%)	Finding
2	Flatten	97.03	0.11	98.89	0.09	Identical Performance
	GlobalAveragePooling2D	96.12	0.25	96.94	0.22	Accuracy Dropped
	GlobalMaxPooling2D	90.73	0.67	91.12	0.58	Accuracy Dropped
	AveragePooling2D	92.95	0.53	93.21	0.47	Accuracy Dropped
	MaxPooling2D	94.78	0.40	95.16	0.36	Accuracy Dropped

**Table 8:** Changing the Loss Function to evaluate the ablation study.

Case Study	Loss Function	Val_Acc (%)	Val_Loss (%)	T_Acc (%)	T_Loss (%)	Finding
3	Categorical Crossentropy	97.03	0.11	98.89	0.09	Identical Performance
	Mean Squared Error	93.77	0.67	94.20	0.58	Accuracy Dropped
	Cosine Similarity	95.82	0.41	96.19	0.37	Accuracy Dropped

**Table 9:** Changing Optimizer and Learning rate to evaluate the ablation study.

Case Study	Optimizer	Learning Rate	Val_Acc (%)	Val_Loss (%)	T_Acc (%)	T_Loss (%)	Finding
4	Adam	0.000001	97.03	0.11	98.89	0.09	Identical Performance
		0.0001	94.50	0.64	95.07	0.47	Accuracy dropped
		0.00001	95.61	0.46	96.22	0.36	Accuracy dropped
		0.001	93.35	0.69	93.98	0.65	Accuracy dropped
		0.01	91.96	0.77	92.35	0.71	Accuracy dropped
	SGD	0.000001	95.12	0.27	95.88	0.21	Accuracy dropped
		0.0001	94.83	0.65	95.15	0.60	Accuracy dropped
		0.00001	94.97	0.63	95.70	0.58	Accuracy dropped
		0.001	90.65	0.80	92.08	0.75	Accuracy dropped
		0.01	92.57	0.72	93.75	0.64	Accuracy dropped
	RMSprop	0.000001	94.57	0.64	94.90	0.49	Accuracy dropped
		0.0001	92.27	0.69	93.20	0.57	Accuracy dropped
		0.00001	93.06	0.58	94.51	0.68	Accuracy dropped
		0.001	92.13	0.72	93.90	0.51	Accuracy dropped
		0.01	91.74	0.80	92.56	0.62	Accuracy dropped
	Nadam	0.000001	95.06	0.25	95.89	0.23	Accuracy dropped
		0.0001	94.26	0.75	95.11	0.68	Accuracy dropped
		0.00001	94.76	0.70	95.32	0.65	Accuracy dropped
		0.001	93.44	0.72	94.81	0.70	Accuracy dropped
		0.01	92.35	0.65	94.20	0.67	Accuracy dropped

## Data Availability

The datasets used for this study can be obtained from the sources mentioned in References [21,22,23,24,25,26,27,28,29,30,31,32,33,34,35,36]. The complete set of code can be also be obtained from the following URL: https://github.com/Shamrat777/Lung-Disease-Classiffication (accessed on 12 April 2022). The Supplementary Material section has the zip file (106 MB) containing the complete runnable code, a sample of the dataset, and a short video and instruction file on how to run the code. Because the dataset provided is a sample of the original one, the result will be different. The sample contains 5000 images, while the original dataset had 80,000 and was over 12 GB in size.

## Supplementary Materials

The following supporting information can be downloaded at: https://www.mdpi.com/article/10.3390/jpm12050680/s1, Video S1: Instruction file on how to run the code. File S1: the complete runnable code. File S2: a sample of the dataset.
