# Supplementary figures and images for: LungNet22: A Fine-Tuned Model for Multiclass Classification and Prediction of Lung Disease Using X-ray Images
Source: J Pers Med. 2022 Apr 24;12(5):680. doi: 10.3390/jpm12050680 (PMC9143659; doi:10.3390/jpm12050680)

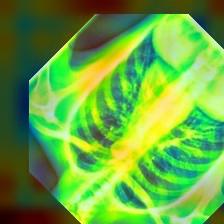

Supplement: Supplementary file 1 [file jpm-12-00680-s001.zip › File S2/control/cam.jpg]

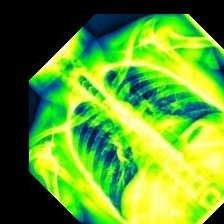

Supplement: Supplementary file 1 [file jpm-12-00680-s001.zip › File S2/control/control00000.jpg]

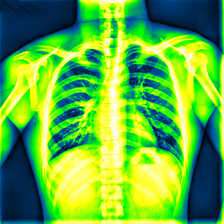

Supplement: Supplementary file 1 [file jpm-12-00680-s001.zip › File S2/control/control00001.png]

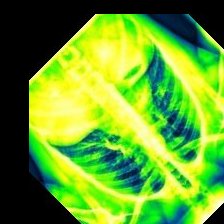

Supplement: Supplementary file 1 [file jpm-12-00680-s001.zip › File S2/control/control00002.jpg]

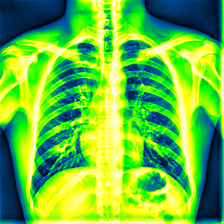

Supplement: Supplementary file 1 [file jpm-12-00680-s001.zip › File S2/control/control00003.png]

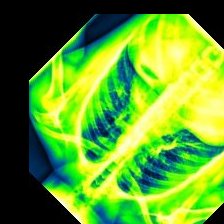

Supplement: Supplementary file 1 [file jpm-12-00680-s001.zip › File S2/control/control00004.jpg]

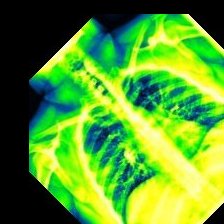

Supplement: Supplementary file 1 [file jpm-12-00680-s001.zip › File S2/control/control00007.jpg]

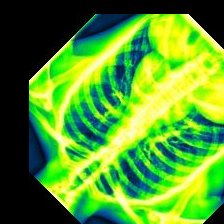

Supplement: Supplementary file 1 [file jpm-12-00680-s001.zip › File S2/control/control00013.jpg]

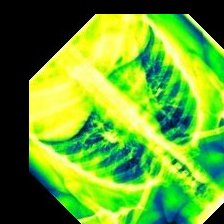

Supplement: Supplementary file 1 [file jpm-12-00680-s001.zip › File S2/control/control00021.jpg]

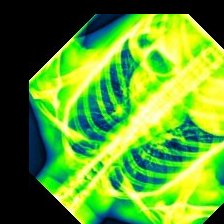

Supplement: Supplementary file 1 [file jpm-12-00680-s001.zip › File S2/control/control00029.jpg]

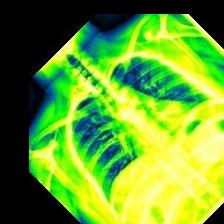

Supplement: Supplementary file 1 [file jpm-12-00680-s001.zip › File S2/control/control00035.jpg]

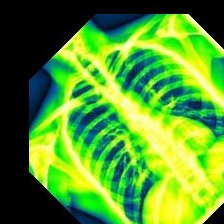

Supplement: Supplementary file 1 [file jpm-12-00680-s001.zip › File S2/control/control00038.jpg]

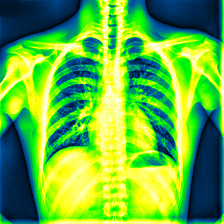

Supplement: Supplementary file 1 [file jpm-12-00680-s001.zip › File S2/control/control00039.png]

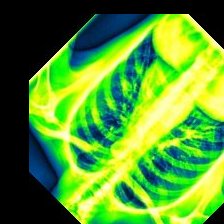

Supplement: Supplementary file 1 [file jpm-12-00680-s001.zip › File S2/control/control00043.jpg]

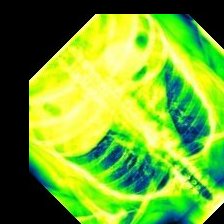

Supplement: Supplementary file 1 [file jpm-12-00680-s001.zip › File S2/control/control00049.jpg]

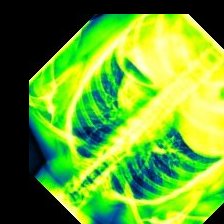

Supplement: Supplementary file 1 [file jpm-12-00680-s001.zip › File S2/control/control00057.jpg]

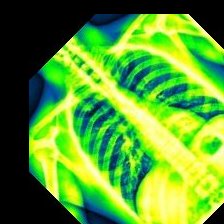

Supplement: Supplementary file 1 [file jpm-12-00680-s001.zip › File S2/control/control00065.jpg]

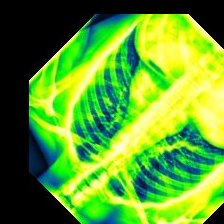

Supplement: Supplementary file 1 [file jpm-12-00680-s001.zip › File S2/control/control00071.jpg]

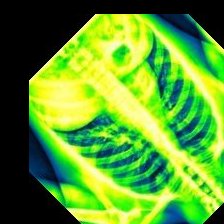

Supplement: Supplementary file 1 [file jpm-12-00680-s001.zip › File S2/control/control00079.jpg]

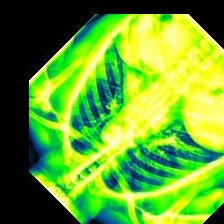

Supplement: Supplementary file 1 [file jpm-12-00680-s001.zip › File S2/control/control00085.jpg]

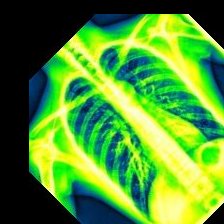

Supplement: Supplementary file 1 [file jpm-12-00680-s001.zip › File S2/control/control00093.jpg]

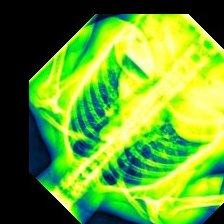

Supplement: Supplementary file 1 [file jpm-12-00680-s001.zip › File S2/control/control00101.jpg]

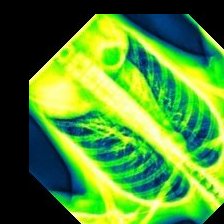

Supplement: Supplementary file 1 [file jpm-12-00680-s001.zip › File S2/control/control00107.jpg]

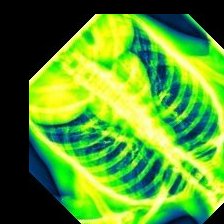

Supplement: Supplementary file 1 [file jpm-12-00680-s001.zip › File S2/control/control00110.jpg]

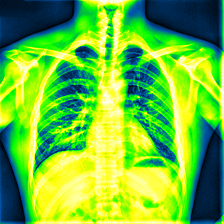

Supplement: Supplementary file 1 [file jpm-12-00680-s001.zip › File S2/control/control00111.png]

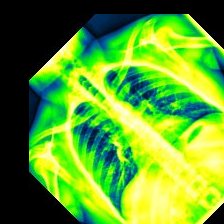

Supplement: Supplementary file 1 [file jpm-12-00680-s001.zip › File S2/control/control00115.jpg]

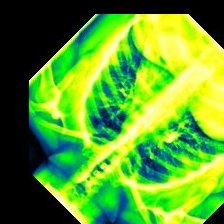

Supplement: Supplementary file 1 [file jpm-12-00680-s001.zip › File S2/control/control00121.jpg]

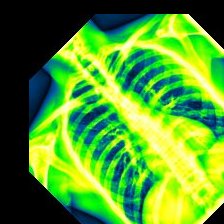

Supplement: Supplementary file 1 [file jpm-12-00680-s001.zip › File S2/control/control00131.jpg]

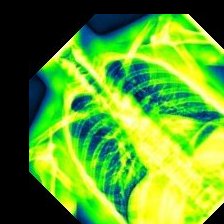

Supplement: Supplementary file 1 [file jpm-12-00680-s001.zip › File S2/control/control00139.jpg]

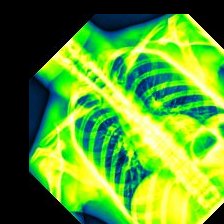

Supplement: Supplementary file 1 [file jpm-12-00680-s001.zip › File S2/control/control00145.jpg]

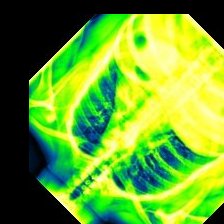

Supplement: Supplementary file 1 [file jpm-12-00680-s001.zip › File S2/control/control00153.jpg]

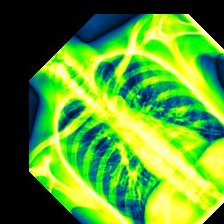

Supplement: Supplementary file 1 [file jpm-12-00680-s001.zip › File S2/control/control00159.jpg]

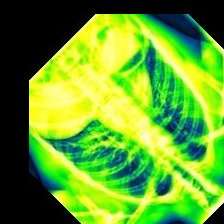

Supplement: Supplementary file 1 [file jpm-12-00680-s001.zip › File S2/control/control00167.jpg]

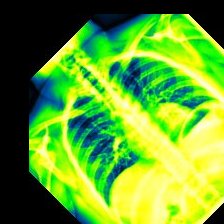

Supplement: Supplementary file 1 [file jpm-12-00680-s001.zip › File S2/control/control00175.jpg]

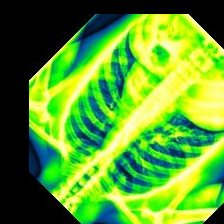

Supplement: Supplementary file 1 [file jpm-12-00680-s001.zip › File S2/control/control00181.jpg]

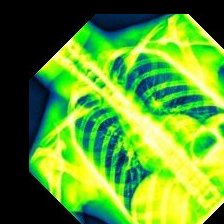

Supplement: Supplementary file 1 [file jpm-12-00680-s001.zip › File S2/control/control00184.jpg]

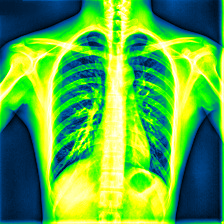

Supplement: Supplementary file 1 [file jpm-12-00680-s001.zip › File S2/control/control00185.png]

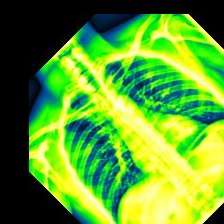

Supplement: Supplementary file 1 [file jpm-12-00680-s001.zip › File S2/control/control00189.jpg]

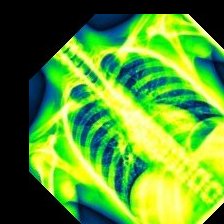

Supplement: Supplementary file 1 [file jpm-12-00680-s001.zip › File S2/control/control00195.jpg]
